# Intraoperative Anesthetic Complications in Pediatric Patients with Mediastinal Mass: A Retrospective Cohort Study

**DOI:** 10.3390/jcm15186987

**Published:** 2026-09-09

**Authors:** Heqi Liu, Jianmin Zhang, Tiehua Zheng, Zeyang Wang

**Affiliations:** Department of Anesthesiology, Beijing Children’s Hospital, Capital Medical University, National Center for Children’s Health, Beijing 100045, China; liuheqi0916@126.com (H.L.);

**Keywords:** anesthetic complications, mediastinal mass, pediatric

## Abstract

**Background:** With advances in diagnostic techniques and therapeutic approaches, updated data on the incidence of intraoperative anesthetic complications in pediatric thoracoscopic mediastinal mass resection are needed. This study aimed to investigate the current incidence, identify potential risk factors, and assess the impact of such complications on postoperative clinical outcomes. **Methods:** We retrospectively reviewed the medical records of all 778 pediatric patients who underwent elective thoracoscopic mediastinal mass resection at our center from January 2023 to December 2025. **Results:** After exclusion of ineligible cases, the incidence of intraoperative anesthetic complications was 28.8% (188/653). Initial clinical symptoms (*p* = 0.007) and one-lung ventilation time (*p* < 0.001) were independently associated with the occurrence of anesthetic complications. Patients who developed intraoperative anesthetic complications had a significantly higher incidence of postoperative pulmonary complications, longer postoperative hospital stay, and longer Intensive Care Unit (ICU) stay compared with those without complications. **Conclusions:** Intraoperative anesthetic complications warrant greater clinical attention because of their high incidence, the difficulty in predicting them from preoperative data alone, and their potential association with adverse postoperative outcomes.

## 1. Introduction

Given that the mass shares the same anatomical space with pulmonary and cardiovascular structures, intraoperative anesthetic complications during pediatric mediastinal mass resection pose a major clinical challenge for anesthesiologists, surgeons, and intensivists [1]. With improved diagnostic rates and the widespread use of tiered diagnosis and treatment systems [2,3,4], it is essential to investigate the current incidence of intraoperative anesthetic complications in children, examine their association with postoperative outcomes and identify potential risk factors.

We conducted a retrospective review of all pediatric patients who underwent elective thoracoscopic mediastinal mass resection at our center from January 2023 to December 2025. Because our center manages a relatively high volume of pediatric mediastinal mass cases annually, this retrospective study is able to include a sizable cohort, which may add useful clinical experience to the existing literature. This study reported the incidence of intraoperative anesthetic complications and analyzed the association with postoperative outcomes and risk factors. We focused on tumor-related characteristics (size, location, compression, and consistency), as well as preoperative laboratory test results and initial clinical presentations. Compared with previous studies, we also examined whether a history of preoperative radiotherapy or chemotherapy and prior thoracic surgery were risk factors for intraoperative anesthetic complications. The findings may have clinical implications. In particular, they could help improve the identification of patients at higher anesthetic risk and inform strategies for risk reduction. They may also contribute to strengthening safety protocols within the tiered diagnosis and treatment system.

## 2. Patients and Methods

This was a single-center, retrospective observational study. The study protocol was approved by the Ethics Committee of Beijing Children’s Hospital ([2026-Y-076-D]) and registered in the Chinese Clinical Trial Registry on 21 May 2026 (ChiCTR2600125081). The study adhered to the Strengthening the Reporting of Observational Studies in Epidemiology (STROBE) reporting guidelines.

Data were extracted from the institutional electronic medical record system (JIAHE) and the anesthesia system (MADISIDON). The study period spanned from January 2023 to December 2025. We screened all pediatric patients (aged < 18 years) who underwent thoracoscopic mediastinal mass resection under general anesthesia during the study period. Data were collected by two independent investigators using a standardized data abstraction form. Discrepancies were resolved by consensus or by adjudication with a third senior investigator.

Inclusion criteria were (1) age ≤ 18 years; (2) scheduled to undergo elective thoracoscopic mediastinal mass resection. Exclusion criteria were: (1) ultrasound-guided mediastinal mass biopsy; (2) concomitant surgical procedures (e.g., partial lung resection, abdominal tumor resection, or spinal tumor resection); (3) missing data exceeding 10% for key variables.

Baseline and Demographic Data included: age, sex, body weight, height, ASA physical status, operative side, robot-assisted. Perioperative variables included: initial clinical symptoms, history of radiotherapy/chemotherapy, history of previous thoracic surgery, comorbidities (e.g., severe pneumonia, mass in other sites, congenital heart disease, congenital syndromes), laboratory tests, imaging findings (standardized tumor volume, mass location, CT attenuation value), operative time, one-lung ventilation time, postoperative pulmonary complication, duration of chest tube drainage, postoperative hospital stay, and ICU stay. The primary outcome was the incidence of intraoperative anesthetic complications, including severe hypotension (MAP decrease > 30% from preoperative baseline), hypoxemia (SpO_2_ ≤ 80%), hypercapnia (EtCO_2_ ≥ 60 mmHg), and other life-threatening adverse events.

Standardized tumor volume [5] was calculated using CT/MRI-derived measurements, as follows: STV = 4/3π × (½mass height × ½mass width × ½mass depth)/patient height (in cm^2^). Postoperative pulmonary complications [6] were defined as the occurrence of any of the following: atelectasis, pneumonia, acute respiratory distress syndrome or pulmonary aspiration.

Data were entered into a de-identified database (Microsoft Excel) for analysis. Continuous variables were assessed for normality using the Shapiro–Wilk test. Data are presented as mean (standard deviation) or median (interquartile range) as appropriate. Categorical variables are presented as frequency (percentage). Between-group comparisons were performed using the *t*-test, Mann–Whitney U test, or chi-squared (χ^2^) test, as appropriate. The Holm–Bonferroni correction was applied to adjust for multiple comparisons. All tests were two-sided, and a *p*-value < 0.05 was considered statistically significant. Risk factors that showed statistically significant differences in these univariate analyses were subsequently entered into a logistic regression model. The significance level for the regression analysis was set at α = 0.05, and the results are presented as odds ratios (ORs) with 95% confidence intervals (CIs) and corresponding *p*-values.

The proportion of missing data for each variable was calculated. Variables with a missing proportion of less than 5% were handled using complete case analysis. For variables with missing data between 5% and 10% (specifically urine output, estimated blood loss (EBL), and time of last chemotherapy), multiple imputation with fully conditional specification (FCS) was performed under the missing at random (MAR) assumption. Variables with missing data exceeding 10% were excluded from the final multivariable analysis to avoid imputation bias.

## 3. Result

**Figure 1:**
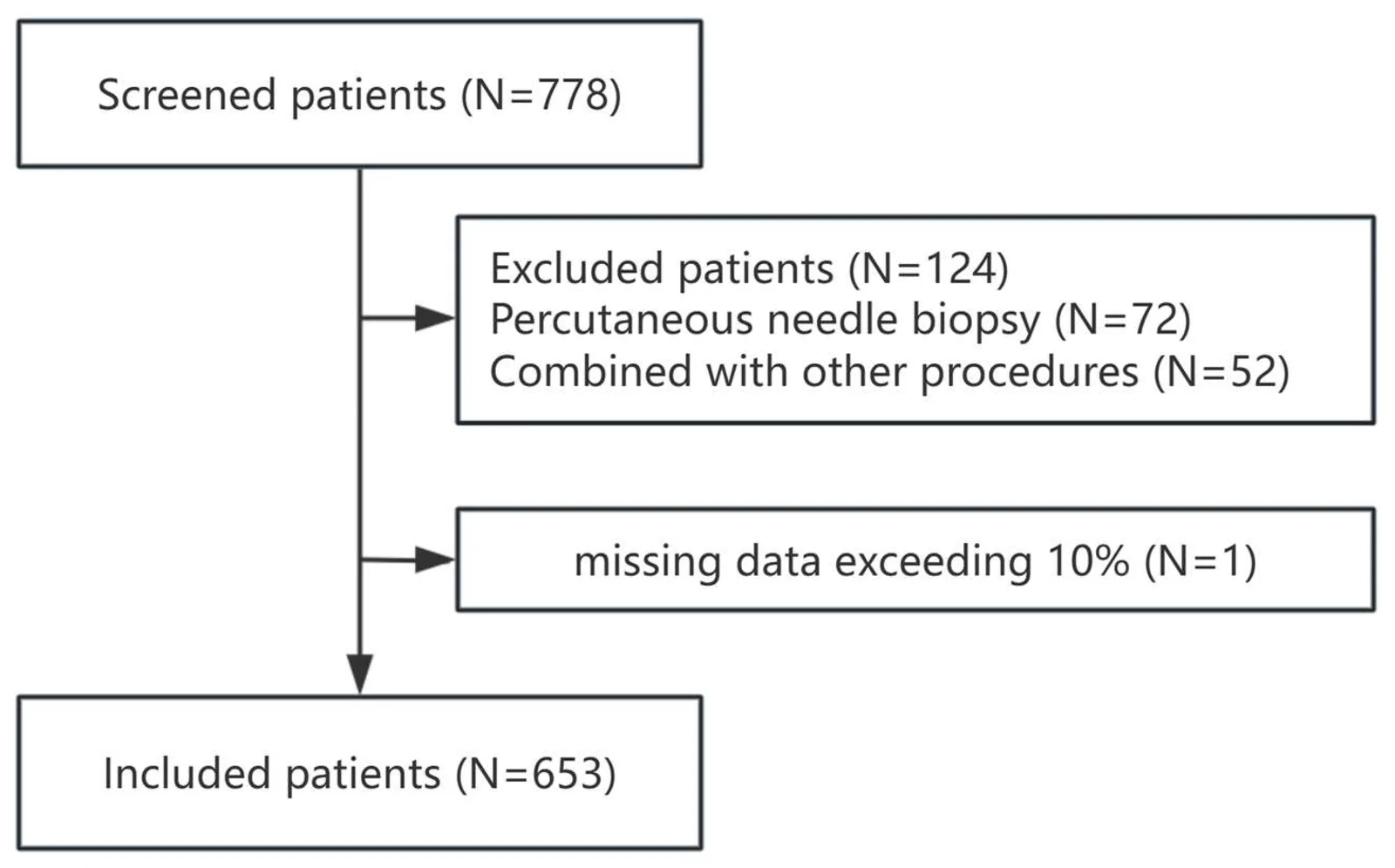
Flow diagram of screened, excluded, and included patients.

From January 2023 to December 2025, a total of 778 pediatric patients underwent elective thoracoscopic mediastinal mass resection at our center. Among them, 72 patients underwent needle biopsy, and 52 patients underwent concurrent surgery at other sites during the operation; these patients were excluded. One additional patient was excluded due to excessive missing key parameters. Consequently, 653 patients were ultimately included in the study (Figure 1). The incidence of anesthetic complications of the included patients is presented in Table 1. With respect to anesthetic complications, severe hypotension occurred in 22% (144/653) of patients, intraoperative hypoxemia in 6% (39/653), and hypercapnia in 7.2% (47/653). The overall incidence of intraoperative anesthetic complications was 28.8% (188/653). No cardiac arrest, malignant arrhythmia, or other life-threatening adverse events were observed in the cohort.

In the comparison between patients with/without intraoperative anesthetic complications, the demographic characteristics of the cohorts are summarized in Table 2; no significant differences were observed. Comparison of preoperative general conditions between the two cohorts (Table 3) showed that the distribution of initial presenting clinical symptoms differed significantly (*p* = 0.001). The preoperative hemoglobin level in the group without anesthetic complications was 126 (119, 134) g/L, which was significantly higher than that in the complication group (*p* = 0.018; median difference [Hodges–Lehmann estimate] 3.00, 95% CI: 0 to 5.00). The standardized tumor volume in the no-complication group was also significantly lower than that in the complication group (*p* < 0.001; median difference −0.13, 95% CI: −0.20 to −0.07). No significant differences were found between the two groups regarding preoperative chemoradiotherapy history, previous thoracic surgery, comorbidities, presence of chest wall deformity, or other preoperative examinations.

In terms of imaging findings (Table 4), the rates of compression of the main airway (*p* = 0.011; OR = 2.19, 95% CI 1.18–4.06), bronchus (*p* < 0.001; OR = 3.32, 95% CI 1.99–5.51), lung tissue (*p* = 0.005; OR = 1.66, 95% CI 1.16–2.39), and great vessels (*p* < 0.001; OR = 2.69, 95% CI 1.66–4.36) were all significantly higher in the group with intraoperative anesthetic complications than in the group without complications. However, mass consistency as diagnosed by CT attenuation values did not differ significantly between the two cohorts (*p* = 0.317).

In the anesthetic complication cohort, the rate of conversion to open thoracotomy was significantly higher than in the non-complication cohort (*p* = 0.011; OR = 1.91, 95% CI 1.16–3.16). Operative time (*p* < 0.001; median difference −34 min, 95% CI −43 to −24), one-lung ventilation time (*p* < 0.001; median difference −23 min, 95% CI −32 to −15), estimated blood loss (*p* < 0.001; median difference 0 mL, 95% CI −1 to 0), and urine output (*p* = 0.012; median difference −20 mL, 95% CI −30 to 0) were all significantly higher/longer in the complication group. No significant differences were observed regarding the use of robotic assistance (*p* = 0.399) or operative side (*p* = 0.671).

To make the results more clinically meaningful and to minimize the interference from collinearity, we dichotomized the variables “initial clinical symptoms” and “mass compression.” Some intraoperative factors (e.g., blood loss, urine output) were not included in the regression analysis. In the multivariable binary logistic regression, initial clinical symptoms (*p* = 0.007) and one-lung ventilation time (*p* < 0.001) were independently associated with anesthetic complications (Table 5).

There were significant differences between the two cohorts in postoperative status, including postoperative pulmonary complications, duration of chest tube drainage, postoperative hospital stay, and ICU stay (Table 6).

## 4. Discussion

The management of mediastinal masses is multidisciplinary, and some pediatric patients require surgical resection. This study focuses specifically on the intraoperative management of children undergoing thoracoscopic resection of mediastinal masses, providing a retrospective analysis primarily from the anesthesia perspective. In this study, the overall incidence of intraoperative anesthetic complications (defined as severe hypotension, hypoxemia, or hypercapnia) was 28.8% (188/653). Previously reported complication rates include 19.6% (11/56) [7], 15% (7/48) [8], 10.9% (5/46) [9], 9.4% (11/117) [10], 7% (6/86) [11]. The incidence observed in our study is substantially higher than those previously reported. Although the definitions of anesthetic complications—i.e., the outcome measures—varied across studies, our clinical experience suggests that this variation has a limited impact on the overall incidence estimation, because children who develop clinically significant anesthesia-related events are very likely to be captured by these outcome indicators. We attribute the higher incidence in our study to several factors. First, our sample size is much larger than those of previous studies. Second, as the largest pediatric thoracic surgery center in China, our center has an extremely high surgical volume but may simultaneously be subject to referral bias, caring for a greater proportion of critically ill patients. Third, previous studies often spanned a decade or longer, whereas our study included only cases from the most recent three years. In summary, the complication rate reported here may better reflect the current standard of care, although it may be slightly higher than the actual population rate.

Anesthesiologists have traditionally regarded large anterior mediastinal masses as higher-risk lesions, and existing studies have consequently focused on this specific subgroup. This selective attention may have introduced subjective bias into the current literature. In the present study, we intentionally selected perioperative variables that are empirically prioritized by anesthesiologists in clinical practice. Nevertheless, the negative findings from our regression analysis may still inform clinical decision-making and may help guide future research.

In the comparison between the two cohorts, significant differences were observed in initial clinical symptoms, preoperative hemoglobin level, standardized tumor volume, conversion to open thoracotomy, operative time, and one-lung ventilation time. However, after regression analysis, initial clinical symptoms and one-lung ventilation time were identified as potential risk factors for complications. Previous studies have indicated that patients who present with orthopnea, severe airway compression symptoms and preoperative tracheal intubation, but not with mass size or location, may experience complications during general anesthesia. [8,11,12] (although some of these studies did not perform regression analysis). However, with the advancement and widespread adoption of prenatal screening and diagnostic techniques in recent years, a considerable number of mediastinal masses are now asymptomatic and are detected incidentally during routine physical examinations. Consequently, high-risk factors for intraoperative anesthetic complications proposed in prior studies [10]—such as orthopnea and upper body edema—are encountered less frequently, underscoring the need for a contemporary risk factor analysis based on the characteristics of the current patient population. Thus, updated evidence indicates that severe intraoperative anesthetic complications are unrelated to preoperative cardiorespiratory symptoms, and that any imaging evidence of airway compression may pose a risk, even in asymptomatic patients [13].

Our results showed that children whose initial presentation was asymptomatic (incidental finding on physical examination) actually had a higher incidence of intraoperative anesthetic complications. This finding is clearly inconsistent with our clinical experience. A review of these cases revealed that many masses were discovered incidentally on CT performed for occasional upper respiratory tract infection; we cannot rule out the possibility that some of these children in fact had recurrent pneumonia. It is also possible that anesthesiologists paid insufficient attention to these seemingly asymptomatic patients. Regardless of the explanation, this finding should prompt reflection: the incidence of intraoperative complications in children without overt clinical symptoms may exceed our expectations, and these patients merit the same degree of vigilance.

We found no significant difference between groups in mass consistency as assessed by CT attenuation values. To our knowledge, few studies have examined this factor in pediatric mediastinal mass resection. This suggests that the incidence of intraoperative anesthetic complications may be similar for cystic and solid masses. This contrasts with our general clinical impression, as we tend to be less cautious with cystic lesions.

In the nonparametric comparison between the two groups, the preoperative hemoglobin level differed significantly; however, the median values in both groups were within the normal range, and this difference was not statistically significant in the multivariable regression analysis. Nevertheless, from a clinical perspective, optimizing the preoperative hemoglobin level, particularly in children with chemotherapy-induced anemia, remains a sound preparatory measure.

Phrenic nerve involvement is of great importance in mediastinal surgery. However, because our study focused on intraoperative anesthetic complications and aimed to identify high-risk patients on the basis of preoperative and intraoperative risk factors, and because phrenic nerve injury is most often diagnosed intraoperatively or postoperatively, this issue was not addressed in the present analysis. We will examine phrenic nerve injury in detail in future studies of postoperative complications.

Significant differences were observed in postoperative pulmonary complications, duration of chest tube drainage, postoperative hospital stay, and ICU stay between children who developed intraoperative anesthetic complications and those who did not. This finding suggests that every effort should be made during intraoperative anesthetic management to minimize the occurrence of hypoxemia, hypotension, and hypercapnia, as these events may be associated with adverse postoperative outcomes.

As a retrospective study, our conclusions are inevitably influenced by the quality of medical record documentation. Moreover, as noted above, as a single-center study, our findings may be subject to bias when generalized to real-world settings. Future prospective studies should evaluate whether optimization of preoperative hemoglobin levels, shortening of operative and one-lung ventilation times where clinically feasible, and improved classification of initial clinical symptoms are associated with fewer intraoperative anesthetic complications and better patient outcomes.

## 5. Conclusions

In summary, the incidence of intraoperative anesthetic complications during pediatric thoracoscopic mediastinal mass resection may have been underestimated in prior reports. In this single-center high-volume cohort, preoperative laboratory results, imaging findings, and standardized tumor volume alone did not provide a robust prediction of intraoperative anesthetic complications after multivariable adjustment. Greater attention should therefore be directed toward intraoperative anesthetic management, particularly oxygenation, hemodynamics, and ventilation. The development of intraoperative anesthetic complications was associated with prolonged hospital stay, a higher incidence of postoperative pulmonary complications, and extended ICU stay, but these associations require cautious interpretation because of the retrospective design and potential outcome-definition overlap.

## Figures and Tables

**Table 1:** Demographic characteristics and incidence of anesthetic complications for all cases.

		N (%)/Median (IQR)
Operative side	Left	273 (41.7%)
	Right	371 (56.8%)
	Both	7 (1.1%)
Difficulty in establishing OLV		14 (2.1%)
Intraoperative (%/min)	Hypotension rate	144 (22%)
	Duration of hypotension	15 (12.5, 17.5)
	Hypoxemia rate	39 (6%)
	Duration of hypoxemia	10 (6.25, 13.75)
	Hypercapnia rate	47 (7.2%)
	Duration of hypercapnia	20 (14, 26)
	Other adverse events	0
Anesthetic complications		188 (28.8%)
Vasoactive agent use rate		29 (4.5%)
Red blood cell transfusion rate		46 (7.0%)
Plasma transfusion rate		30 (4.6%)

**Table 2:** Demographic characteristics.

		Complications		*p*
		None *n* = 465	Yes *n* = 188	
Sex	Male	254 (54.6%)	96 (51.1%)	0.409
	Female	211 (45.4%)	92 (48.9%)	
Age		7 (4, 10)	6 (3, 10)	0.22
Weight		23.4 (17.0, 37.8)	22.1 (14.6, 40.0)	0.178
Height		123 (106, 143)	118 (95, 148)	0.104
ASA	II	427 (91.8%)	161 (85.6%)	0.086
	III	37 (8%)	26 (13.8%)	
	IV	1 (0.2%)	1 (0.2%)	

**Table 3:** Preoperative conditions.

		Complications		*p*	95%CI
		None *n* = 465	Yes *n* = 188		
History of chemoradiotherapy	Yes	53 (11.4%)	29 (15.4%)	0.160	1.42 (0.87, 2.31)
	No	412 (88.6%)	159 (84.6%)		
History of thoracic surgery	Yes	51 (11%)	26 (13.8%)	0.305	1.30 (0.79, 2.16)
	No	414 (89%)	162 (86.2%)		
Last chemotherapy (Days)		20 (14, 50)	20 (10, 30)	0.178	5 (−1.00, 10.00)
Comorbidities					
None		415 (89.2%)	166(88.3%)	0.405	NA
Severe pneumonia		6 (1.3%)	1 (0.5%)		
Tumors of other sites		29 (6.2%)	10 (5.3%)		
Congenital heart disease		5 (1.1%)	6 (3.2%)		
Hormonal secretion disorder		2 (0.4%)	1 (0.5%)		
Other syndromes		12 (1.7%)	4 (2.1%)		
Chest wall deformity		2 (0.4%)	2 (1.1%)	0.347	2.49 (0.35, 17.80)
Initial Clinical Symptoms:					
**None**		**69 (14.8%)**	**43 (22.9%)**	**0.001**	NA
Dry cough		67 (14.4%)	18 (9.6%)		
Recurrent pneumonia		210 (45.2%)	66 (35.1%)		
Chest pain		29 (6.2%)	20 (10.6%)		
Fever		45 (9.7%)	10 (5.3%)		
Neural symptoms		9 (1.9%)	12 (6.4%)		
Dyspnea		22 (4.7%)	15 (8%)		
Endocrine disorders		3 (0.6%)	2 (1.1%)		
Gastrointestinal symptoms		11 (2.4%)	2 (1.1%)		
Alb (g/L)		43.6 (41.9, 45.4)	43.5 (41.4, 45.0)	0.227	0.30 (−0.20, 0.80)
WBC (×10^9^/L)		6.81 (5.4, 8.2)	6.86 (5.7, 8.5)	0.336	−0.19 (−0.59, 0.21)
**Hb (g/L)**		**126 (119, 134)**	**124 (115, 131)**	**0.018**	**3.00 (0, 5.00)**
Plt (×10^9^/L)		311 (258, 367)	310 (251, 389)	0.986	0 (−0.10, 1.20)
APTT (s)		35.3 (33.1, 37.7)	34.7 (32.0, 37.5)	0.105	0.6 (−0.10, 1.20)
**STV(cm^2^)**		**0.180 (0.073, 0.600)**	**0.374 (0.114, 1.407)**	**<0.001**	**−0.13 (−0.20, −0.07)**

Alb, albumin; WBC, White blood cell; Hb, Hemoglobin; Plt, platelet count; APTT, Activated partial thromboplastin time; STV, standardized tumor volume. Bold values are statistically significant (*p* < 0.05).

**Table 4:** Imaging examinations and intraoperative conditions.

		Complications		*p*	95%CI
		**None *n* = 465**	**Yes *n* = 188**		
**Compression**	**Main airway**	**24 (5.2%)**	**20 (10.6%)**	**0.011**	**2.19 (1.18, 4.06)**
	**Bronchial**	**32 (6.9%)**	**37 (19.7%)**	**<0.001**	**3.32 (1.99, 5.51)**
	**Lung tissue**	**122 (26.2%)**	**70 (37.2%)**	**0.005**	**1.66 (1.16, 2.39)**
	**Great vessels**	**40 (8.6%)**	**102 (54.3%)**	**<0.001**	**2.69 (1.66, 4.36)**
Anterior mediastinal mass		102 (21.9%)	54 (28.7%)	0.065	1.43 (0.98, 2.11)
Posterior mediastinal mass		329 (70.7%)	120 (63.8%)	0.084	0.73 (0.51, 1.04)
Methods for OLV					
Endotracheal with bronchial blocker		311 (66.9%)	109 (58%)	0.197	0.96 (0.42, 2.21)
Double-lumen endotracheal tube		93 (20%)	50 (26.6%)		
Endobronchial intubation		28 (6.0%)	12 (6.4%)		
Laryngeal mask with bronchial blocker		10 (2.2%)	3 (1.6%)		
Two-lung ventilation		23 (4.9%)	14 (7.4%)		
Difficulty in establishing OLV		7 (1.5%)	7 (3.7%)	0.076	2.53 (0.88, 7.32)
CT value	Lipoma	20 (4.3%)	12 (6.4%)	0.317	NA
(HU)	Cystic mass	62 (13.3%)	34 (18.1%)		
	Cystic, solid mass	80 (17.2%)	31 (16.5%)		
	Solid mass	187 (40.2%)	69 (36.7%)		
Robot-assisted	Yes	67 (14.4%)	32 (17%)	0.399	1.21 (0.77, 1.93)
**Conversion to thoracotomy**		**42 (9%)**	**30 (16%)**	**0.011**	**1.91 (1.16, 3.16)**
**Operative time (min)**		**82 (55, 124)**	**121 (82, 165)**	**<0.001**	**−34 (−43, −24)**
**OLV time (min)**		**69 (45, 105)**	**91 (61, 135)**	**<0.001**	**−23 (−32, −15)**
Operative side	Left	194 (41.7%)	79 (42%)	0.671	NA
	Right	263 (56.6%)	108 (57.4%)		
	Both	6 (1.3%)	1 (0.5%)		
**Blood loss (mL)**		**2 (2, 5)**	**2 (2, 10)**	**<0.001**	**0 (−1, 0)**
**Urine output (mL)**		**80 (30, 150)**	**100 (50, 200)**	**0.012**	**−20 (−30, 0)**

Bold values are statistically significant (*p* < 0.05).

**Table 5:** Multivariable binary logistic regression.

	*p*	OR	95%CI
Initial Clinical Symptoms (None)	0.007	1.847	1.181–2.889
Hb (g/L)	0.283	1.001	0.999–1.002
Mass Compression	0.142	0.758	0.523–1.097
STV (cm^2^)	0.067	1.039	0.997–1.083
**OLV time (min)**	**<0.001**	**1.006**	**1.003–1.009**
Conversion to thoracotomy	0.401	0.790	0.456–1.369

Bold values are statistically significant (*p* < 0.05).

**Table 6:** Postoperative Status.

	Complications		*p*
	**None *n* = 465**	**Yes *n* = 188**	
**Postoperative Pulmonary Complication**	**82 (17.6%)**	**64 (34.0%)**	**<0.001**
**Duration of Chest Tube Drainage (Day)**	**4 (3, 5)**	**5 (4, 6)**	**<0.001**
**Postoperative Hospital Stay (Day)**	**5 (5, 6)**	**6 (5, 8)**	**<0.001**
Postoperative ICU Transfer	15 (3.2%)	17 (9.0%)	0.002
ICU Stay (Day)	0	0	0.003

Bold values are statistically significant (*p* < 0.05).

## Data Availability

The data were obtained from a medical records database under the supervision of the Ethics Committee and the Data Management Committee. Data are not available for ethical reasons.
